# The Peptide A-3302-B Isolated from a Marine Bacterium *Micromonospora* sp. Inhibits HSV-2 Infection by Preventing the Viral Egress from Host Cells

**DOI:** 10.3390/ijms23020947

**Published:** 2022-01-15

**Authors:** Sanya Sureram, Irene Arduino, Reiko Ueoka, Massimo Rittà, Rachele Francese, Rattanaporn Srivibool, Dhanushka Darshana, Jörn Piel, Somsak Ruchirawat, Luisa Muratori, David Lembo, Prasat Kittakoop, Manuela Donalisio

**Affiliations:** 1 Chulabhorn Research Institute, Kamphaeng Phet 6 Road, Laksi, Bangkok 10210, Thailand; sanya@cri.or.th (S.S.); somsak@cri.or.th (S.R.); 2 Department of Clinical and Biological Sciences, University of Turin, Regione Gonzole 10, 10043 Orbassano, Italy; irene.arduino@unito.it (I.A.); massimo.ritta@unito.it (M.R.); rachele.francese@unito.it (R.F.); david.lembo@unito.it (D.L.); 3 Institute of Microbiology, ETH Zurich, Vladimir-Prelog-Weg 4, 8093 Zurich, Switzerland; rueoka@kitasato-u.ac.jp (R.U.); jpiel@ethz.ch (J.P.); 4 Institute of Marine Science, Burapha University, Chonburi 20130, Thailand; jeme.rattanaporn@gmail.com; 5 Program in Chemical Sciences, Chulabhorn Graduate Institute, Chulabhorn Royal Academy, Kamphaeng Phet 6 Road, Laksi, Bangkok 10210, Thailand; dhanushkadsilva@gmail.com; 6 Center of Excellence on Environmental Health and Toxicology (EHT), OPS, Ministry of Higher Education, Science, Research and Innovation, Bangkok 10210, Thailand; 7 Department of Clinical and Biological Sciences, Neuroscience Institute of the “Cavalieri Ottolenghi” Foundation (NICO), University of Turin, 10043 Orbassano, Italy; luisa.muratori@unito.it

**Keywords:** rare actinomycete, *Micromonospora*, marine natural products, natural antiviral products, egress inhibitor, herpes simplex virus type 2

## Abstract

Herpesviruses are highly prevalent in the human population, and frequent reactivations occur throughout life. Despite antiviral drugs against herpetic infections, the increasing appearance of drug-resistant viral strains and their adverse effects prompt the research of novel antiherpetic drugs for treating lesions. Peptides obtained from natural sources have recently become of particular interest for antiviral therapy applications. In this work, we investigated the antiviral activity of the peptide A-3302-B, isolated from a marine bacterium, *Micromonospora* sp., strain MAG 9-7, against herpes simplex virus type 1, type 2, and human cytomegalovirus. Results showed that the peptide exerted a specific inhibitory activity against HSV-2 with an EC_50_ value of 14 μM. Specific antiviral assays were performed to investigate the mechanism of action of A-3302-B. We demonstrated that the peptide did not affect the expression of viral proteins, but it inhibited the late events of the HSV-2 replicative cycle. In detail, it reduced the cell-to-cell virus spread and the transmission of the extracellular free virus by preventing the egress of HSV-2 progeny from the infected cells. The dual antiviral and previously reported anti-inflammatory activities of A-3302-B, and its effect against an acyclovir-resistant HSV-2 strain are attractive features for developing a therapeutic to reduce the transmission of HSV-2 infections.

## 1. Introduction

*Herpesviridae* is a large family of DNA viruses that includes eight species of pathogenic human viruses. Human herpesviruses are ubiquitous, affecting 60–95% of the population worldwide [1,2]. These pathogens can cause a lytic infection with acute clinical manifestations, but a relevant feature of herpesvirus infection is the ability to establish a latent lifelong infection in the host. The latent virus can undergo reactivation determining a recurrent lytic infection in the site of the primary infection or a severe disseminated infection. Human herpesviruses exhibit a spectrum of clinical manifestations, generally self-limiting or asymptomatic. However, they can cause severe disease in immunocompromised patients, such as those with AIDS or transplant patients. Transmission occurs mainly through direct close contact with body secretions containing infectious virus. Herpesvirus infections can also be transmitted vertically, from the mother to the fetus/newborn, leading to life-threatening illness and long-term sequelae in the infant.

Two types of human herpes simplex viruses (HSV) are known: HSV-1, which preferentially infects orolabial tissues, and HSV-2, which is almost exclusively sexually transmitted and infects preferentially genital mucosae. Genital herpes, caused mostly by HSV-2, affects more than 400 million persons worldwide, and its prevalence is rising due to sexual promiscuity, lack of safe sex practices, and high frequency of asymptomatic but infectious cases [3,4,5,6]. Moreover, HSV-2 genital infection has been strongly associated with HIV infections, primarily due to the three-fold increase in the risk of acquiring HIV in case of an HSV-2 established infection [7]. Another member of the herpetic family is human cytomegalovirus (HCMV), with a high prevalence worldwide (30–100%) [8]. HCMV infection is the most common congenital infection transmitted by maternal primary exposure or reactivation; it can cause sensorineural hearing loss and neurodevelopmental disabilities in children.

Nucleoside analogues (i.e., Acyclovir, Valacyclovir, and Famciclovir for HSV; Ganciclovir and Foscarnet for HCMV) are the most well-known and used class of approved antiherpetic drugs, acting as competitive inhibitors of herpesvirus DNA polymerase. Although these antivirals can decrease the severity of the disease and shorten outbreaks, they are not active on the latent nonreplicating form of herpesviruses. Furthermore, due to their wide use and prolonged administration, drug-resistant viral strains are common nowadays, particularly in immunocompromised patients undergoing long-term therapy [9,10]. For instance, acyclovir-resistant HSV-2 strains have been observed in 0.3% of immunocompetent patients, whereas their prevalence reaches 3–6% in immunocompromised patients and up to 5% in HIV-positive patients [11]. These limitations suggest the need to identify new antiviral drugs with a different mechanism than nucleoside analogues to counteract the rising resistance issue.

In recent years, peptides emerged as a promising class of bioactive molecules for various biomedical applications, resulting in more than 60 approved peptide drugs and numerous clinical trials. Among other applications, peptides have shown interesting antiviral properties, including the potential role of treating severe acute respiratory syndrome coronavirus 2 (SARS-CoV-2) infections [12,13,14,15,16]. Some peptide-based antiviral therapies have already been approved for clinical indication, such as fusion inhibitor Enfuvirtide for the Human immunodeficiency virus (HIV) and protease inhibitors Boceprevir and Telaprevir for Hepatitis C virus (HCV) [17,18,19]. Recently, two potent antiviral derivatives of Boceprevir and Telaprevir were designed based on the inhibition of main protease (M^pro^) of SARS-CoV-2 and their administration in SARS-CoV-2 infected rats could considerably reduce lung viral loads and lung lesions [20].

Natural products are potential sources for drug development, and a number of drugs have been derived or inspired from natural products [21,22]. They also provide pharmacophores for medicinal chemistry [23]. In particular, antiviral peptides have been recently isolated from various natural sources and showed promising antiviral activity against different human viruses [24]. The marine bacteria belonging to the genus *Micromonospora* have been previously investigated for the various properties of their peptidic metabolites, such as cytotoxic and antibacterial activities [25,26,27]. However, the antiviral activity of a natural compound isolated from *Micromonospora* sp. has never been demonstrated. Herein, we report the isolation of a natural peptide, namely A-3302-B, from a marine bacterium, *Micromonospora* sp. strain MAG 9-7, and the study of its antiviral properties against selected members of the *Herpesviridae* family (HSV-1, HSV-2 and HCMV). The anti-HSV-2 inhibitory effect was explored to clarify its mechanism of action.

## 2. Results and Discussion

### 2.1. Isolation of the Peptide A-3302-B from the Marine Bacterium Micromonospora sp. MAG 9-7

**Figure 1:**
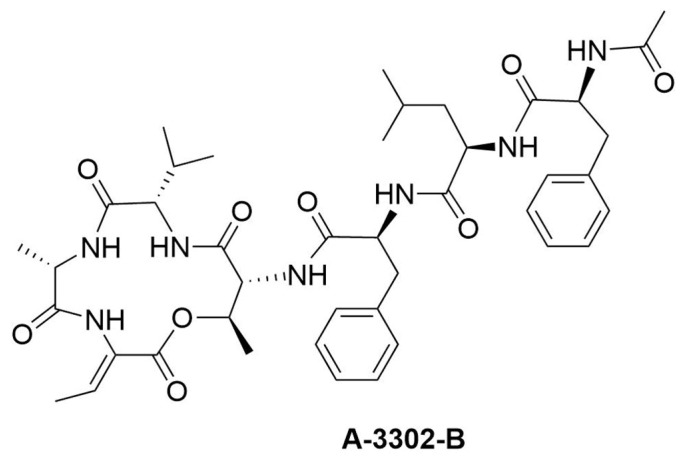
Chemical structure of peptide A-3302-B.

A crude extract of a marine bacterium, *Micromonospora* sp. MAG 9-7, was purified by Sephadex LH-20 column chromatography, and the peptide A-3302-B was identified (Figure 1). The peptide was previously isolated from other bacteria and referred to as A-3302-B, TL-119, or ASP-1 [28,29]. The ^1^H and ^13^C NMR spectra of A-3302-B showed typical resonances for peptides (Figures S1 and S2), and NMR data are identical in all respects to those in the literature [30]. The HRESI-MS spectrum of A-3302-B (Figure S3) showed the ion at *m*/*z* 804.4256 [M + H]^+^, calculated for C_42_H_58_N_7_O_9_, 804.4296. The MS/MS spectrum of A-3302-B (Figure S4) confirmed the sequence of amino acids of this peptide. In the above-mentioned previous works, the peptide, isolated from other bacteria, exhibited antistaphylococcal activity. Furthermore, it was previously reported to have anti-inflammatory activity, being a transient receptor potential vanilloid-1 (TRPV-1) antagonist [31], and a patent was filed for this activity [32].

### 2.2. Antiviral Activity of A-3302-B against Herpetic Viruses

**Figure 2:**
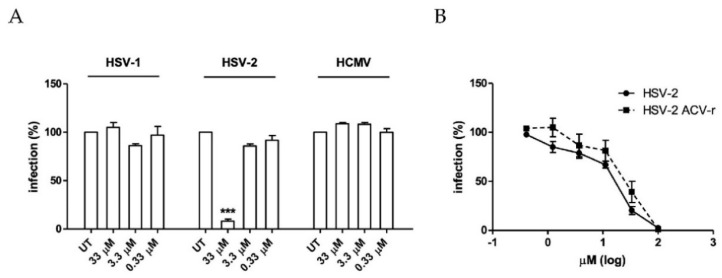
The antiherpetic activity of A-3302-B. (**A**) The antiviral activity of A-3302-B was evaluated by plaque reduction assays for HSV-1 and HSV-2 and by focus reduction assay for HCMV, treating cells with A-3302-B at three fixed doses before, during and after infection. One-way analysis of variance (ANOVA) was used to compare treated and control samples. *** = *p*_ANOVA_ < 0.001. (**B**) The antiviral activity of the peptide against wildtype HSV-2 and acyclovir-resistant HSV-2 (HSV-2 ACV-r) was evaluated by plaque reduction assay treating cells before, during, and after infection with increasing concentrations of compound (0.4 to 100 μM). The per cent infection (%) was calculated by comparing A-3302-B treated and DMSO-treated samples. Results from three independent experiments are reported as the mean and standard error of the mean (SEM).

To investigate the antiviral activity of A-3302-B against herpetic viruses, a preliminary screening was performed against HSV-1, HSV-2, and HCMV. To this end, we carried out plaque reduction assays for HSV-1 and HSV-2 and focus reduction assays for HCMV, treating cells with the compound at three doses (33, 3.3, 0.3 μM) before, during, and after infection. As shown in Figure 2A, A-3302-B was active against HSV-2 in a dose-dependent manner. Notably, A-3302-B did not exhibit inhibitory activity against the other selected members of the *Herpesviridae* family, i.e., HSV-1 and HCMV, indicating that its antiviral activity is specific to HSV-2. The difference in sensitivity to antiviral agents of HSV-1 and HSV-2, although genetically and structurally similar, may be ascribed to different viral strains or cell types used in the antiviral assays, as previously reported [33].

The inhibitory activity of A-3302-B against HSV-2 was then confirmed, testing a wider range of concentrations (0.4 to 100 μM) by plaque reduction assay to generate dose–response curves. The compound exerted a marked dose-dependent activity against HSV-2, with an EC_50_ value of 14.22 μM and an EC_90_ value of 79.64 μM (Figure 2B). Notably, the antiviral effect of the compound was not a consequence of cytotoxicity since it did not significantly reduce cell viability at any concentration used in the antiviral assays, exhibiting a CC_50_ value of 396.8 μM (Figure S6).

Previous studies evidenced marine natural sources of antiherpetic peptides. For instance, Boas et al. demonstrated the anti-HSV-1 activity of the peptide Pa-MAP from the polar fish *Pleuronectes americanus*, and a recent work investigated the anti-HSV-1 activity of peptides from the deep-sea-derived fungal strain *Simplicillium obclavatum* sp. [34,35]. A-3302-B exerted an antiviral activity with an EC_50_ value in the same range as those reported.

The widespread emergence of HSV-2 strains resistant to available drugs is limiting the therapeutic options. Therefore, the peptide was assessed in vitro against a laboratory strain of HSV-2 resistant to acyclovir (HSV-2 ACV-r) previously generated in our laboratory [36]. It was active against HSV-2 ACV-r, exhibiting an EC_50_ value of 24.92 µM, similar to the one obtained for the acyclovir-sensitive strain (Figure 2B). This finding suggested that A-3302-B may act through an alternative antiviral mechanism to that of acyclovir.

### 2.3. Investigation of A-3302-B Mechanism of Action

We first explored whether the antiviral activity of A-3302-B was exerted via the direct inactivation of HSV-2 virus particles. To this end, we incubated for 2 h at 37 °C a mixture of 10^5^ PFU of HSV-2 and A-3302-B at the EC_90_ dose, which was then titrated on cells at noninhibitory concentrations of the compound. No difference in viral titers of treated and control samples was observed, indicating that the compound did not exert intrinsic virucidal activity (Figure S7). This finding contrasted with many reports in the literature in which natural peptides showed direct inactivation of HSV particles by disrupting the viral envelope [37,38,39,40,41].

**Figure 3:**
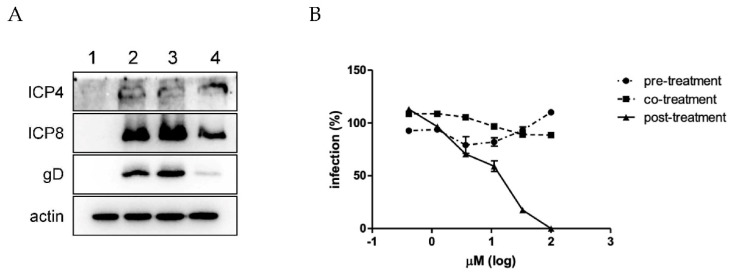
Investigation of the mechanism of action of A-3302-B against HSV-2. (**A**) The effect of A-3302-B treatment on the expression of HSV-2 proteins was evaluated by immunoblotting. Vero cells were infected (MOI 1 PFU/cell) and treated before, during, and after infection with A-3302-B or ACV. A total of 4 hpi whole-cell extracts were prepared as described in the Section 3 and analyzed using antibodies directed against the following viral proteins: ICP4, ICP8, and gD. Actin was used as an internal control. 1: uninfected untreated cells. 2: infected untreated cells. 3: infected cells treated with A-3302-B at the EC_90_ dose. 4: infected cells treated with ACV at 15 μM. (**B**) Time-of-addition assays were carried out, adding the compound (0.4 to 100 μM) to cells for 2 h before infection (pre-treatment), for 2 h during infection (co-treatment) or 24 h after removal of virus inoculum (post-treatment), as described in the Section 3. Plaque formation was assessed 24 h after infection. The percent infection (%) was calculated by comparing A-3302-B treated and DMSO-treated samples. Results are reported as mean and SEM for three independent experiments.

The intracellular HSV replicative cycle is based on the sequential expression of the viral immediate–early (IE), early (E) and late (L) genes. In particular, IE genes encode regulatory proteins, such as ICP4, E genes encode proteins needed for DNA replication, such as ICP8, and L genes encode structural viral proteins, such as glycoprotein gD. Therefore, to investigate which phase of HSV gene expression was inhibited by the peptide, we analyzed the expression of IE, E, and L viral proteins (ICP4, ICP8, and gD, respectively) after A-3302-B treatment by immunoblot analysis. Treatment with ACV, the drug of choice for antiherpetic therapy, was used as a control. ACV acts in a phase of the viral replicative cycle prior to L gene expression, as it inhibits the replication of the viral genome. As expected, the immunoblotting results showed that ACV inhibited the expression of the L protein gD (Figure 3A). Interestingly, A-3302-B did not inhibit the expression of any viral proteins at 4 hpi, and this result was confirmed at 16 hpi (data not shown). This finding indicated that the compound inhibits a late step of the viral replicative cycle occurring after viral genome replication and L protein expression (e.g., virus assembly or egress from the infected host cells).

**Figure 4:**
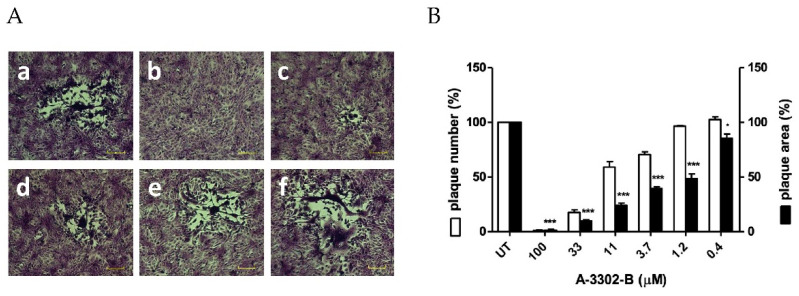
Effect of A-3302-B on cell-to-cell spread of HSV-2. (**A**) Representative HSV-2 plaques in Vero cells observed in the post-treatment assay are reported for DMSO-treated control (**a**) and for the following concentrations of A-3302-B: (**b**) 100 µM, (**c**) 33 µM, (**d**) 3.7 µM, (**e**) 1.2 µM, (**f**) 0.4 µM. Scale bar, 100 μm. (**B**) The bar chart shows the percentage of HSV-2 plaque count (white) and plaque area (black) of treated wells compared to that of control wells as a function of the concentration of A-3302-B in the post-treatment assay. Treated and control samples were compared with one-way ANOVA. * = *p*_ANOVA_ < 0.05; *** = *p*_ANOVA_ < 0.001. The pictures and bar chart are representative of ≥10 plaques per condition. UT: untreated.

To confirm our hypothesis, time-of-addition assays were performed, adding the compound to the cells only before, during, or after infection. As reported in Figure 3B, when added to cells before the infection (pre-treatment) or with the virus inoculum in the first 2 h of infection (co-treatment, Figure 3B), A-3302-B was not active, suggesting that early cell membrane–virus interactions were not affected by the peptide. These findings were further confirmed by more stringent assays (i.e., attachment assay and entry assay) (Figure S8). Instead, A-3302-B was active only when added after infection (post-treatment, Figure 3B), with an EC_50_ value of 11.41 μM, supporting the hypothesis that A-3302-B affects later steps of the HSV-2 replicative cycle. The bright-field optical microscope examination of cells treated after infection showed that A-3302-B reduced in a dose-dependent manner not only the viral plaque number but also the viral plaque area (Figure 4A,B). Notably, no viral plaques were detected at the highest tested concentration, i.e., 100 μM. These data suggested that the peptide inhibits cell-to-cell spread, and this could be a result of inhibiting the production of infectious viral progeny or blocking the exit of the virus from the cell.

**Figure 5:**
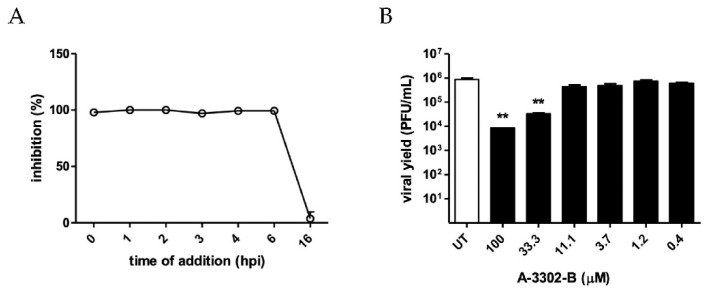
Effect of A-3302-B on late steps of HSV-2 replicative cycle. (**A**) The inhibitory activity of A-3302-B against HSV-2 was assessed at different time points postinfection. Briefly, Vero cells were treated with the compound at the EC_90_ dose at 0, 1, 2, 3, 4, 6, and 16 h postinfection. The percent inhibition (%) was determined by comparing A-3302-B treated and DMSO-treated samples. Results are reported as mean and SEM for three independent experiments. (**B**) The effects of A-3302-B on HSV-2 progeny production were assessed by virus yield reduction assay. Vero cells were infected with HSV-2 for 2 h and subsequently treated with increasing concentrations of the compound (0.4 to 100 μM). When control wells exhibited extensive viral cytopathic effect, the samples were collected and titrated. Viral titers are expressed as PFU/mL and shown as mean and SEM for three independent experiments. Treated and control samples were compared with one-way ANOVA. ** = *p*_ANOVA_ < 0.01.

Interestingly, when the peptide was administered postinfection at different time points (from 1 h to 16 h after removal of the virus inoculum), the inhibitory effect of A-3302-B was maintained up to 6 h postinfection (Figure 5A). This finding further points out that the peptide exerts its inhibitory activity during the late events of HSV-2 replication occurring at least 6 h postinfection, and this action takes place rapidly after the administration of A-3302-B. Furthermore, this result is significant given a possible therapeutic application of A-3302-B for the treatment of an established local HSV-2 infection.

Next, we evaluated whether the compound was able to affect the production of infectious viral progeny. Specifically, we performed a virus yield reduction assay, treating cells after infection with increasing concentration of A-3302-B (0.4 to 100 μM) for 24 h and then performing titration of the harvested virus samples. As reported in Figure 5B, A-3302-B significantly reduced the HSV-2 titer at high tested concentrations (100 μM and 33 μM), exhibiting an EC_50_ value of 11.31 μM. An additional assay was performed at the highest tested dose (100 μM) as the aforementioned virus yield reduction assay but titrating separately the infectious virus located intracellularly and released in the culture supernatant. As shown in Table 1, A-3302-B determined a significant decrease of viral titer in the culture supernatant of the treated cells (6.00 × 10^0^ PFU) compared with the untreated control (1.01 × 10^3^ PFU). The intracellular viral titer was reduced 10-fold compared with the untreated control. These results can be ascribed to the putative mechanism of action of the peptide, which allows the production of infectious viral particles in cells originally infected (before treatment), accounting for the intracellular viral titer in treated samples, but hampers the egress of the virus from the infected host cells resulting in the reduction of the extracellular viral titer.

**Figure 6:**
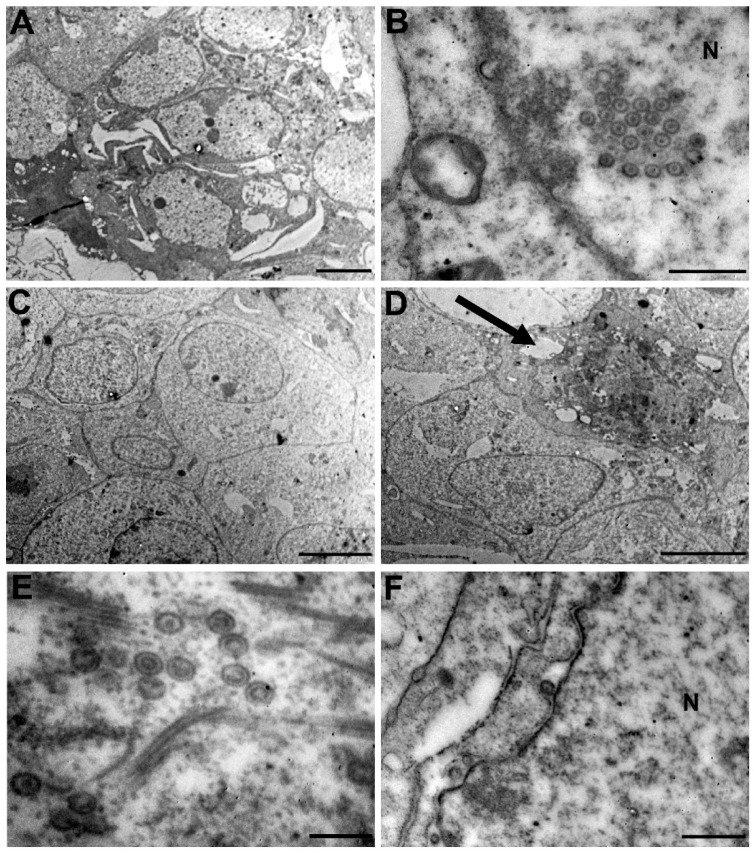
Transmission electron microscopy analysis of HSV-2 infected Vero cells treated with A-3302-B. Vero cells were infected with HSV-2 and then treated with A-3302-B (100 µM) or overlaid with the control medium. At 16 hpi, cells were harvested and prepared for electron microscopy. Representative images of infected Vero cells are reported at low magnification (**A**) and higher magnification (**B**). Representative images of infected treated Vero cells are reported at low magnification ((**C**) healthy cell monolayer; (**D**) single infected cell (black arrow)) and higher magnification (**E**,**F**). N: nucleus. (**A**,**C**,**D**) Scale bar, 5 µm; (**B**,**F**) Scale bar, 0.5 µm; (**E**) Scale bar, 0.2 µm.

Finally, transmission electron microscopy (TEM) analysis was performed to further investigate the effect of A-3302-B on infected cells. To this aim, Vero cells were infected with HSV-2 and then treated with the peptide. Cells were subsequently harvested 16 hpi and processed for TEM procedure as described in the Section 3. As shown in Figure 6A,B, untreated infected cells showed an advanced cytopathic effect due to multiple cycles of infection, and the new viral progeny was detectable at a different stage of the replicative cycle in both nucleus and cytoplasm. On the other hand, after treatment, most of the cells did not evidence signs of infection, maintaining healthy cellular morphology with intact organelles such as a well-defined nucleus, as shown in Figure 6C. Of note, few isolated infected cells were detected, in which fully developed viral particles were visible (Figure 6D–F). These data support the previously reported results that show the peptide, added after infection, exerted its antiviral action blocking the egress of virions from infected cells, thus preventing the viral spread and an extensive cytopathic effect.

The literature reports anti-HSV peptides endowed with different mechanisms of action. For instance, a short amphibian peptide temporin B demonstrated both virucidal activity and inhibitory activity in different phases of the HSV-1 life cycle, including the entry of the virus into the host cell and the subsequent postinfection phase [41]. In another work, the authors identified synthetic peptides derived from a capsid protein of HSV-1, capable of inhibiting the membrane-budding activity of the nuclear egress complex (NEC) [42]. The sequence of A-3302-B differs from that of the synthetic peptidic inhibitors of the NEC complex.

Concerning the current study, A-3302-B turned out to be a late inhibitor of HSV-2 infection, most likely preventing the egress of newly produced viruses from host cells. This putative mechanism of action is supported by the following experimental evidence: (i) A-3302-B is active when administered after the viral inoculum, and the inhibition of HSV-2 replication is maintained up to 6 h postinfection; (ii) The expression of viral proteins and the assembly of infectious viral particles are preserved; (iii) The cell-to-cell spread of virions is inhibited in treated cellular monolayers; (iv) The release of extracellular free virus in culture supernatant is strongly reduced. Further work needs to be carried out to identify the cellular or viral molecular targets involved in A-3302-B inhibition of HSV-2 infection. Notably, given the putative mechanism of action of A-3302-B, the difference in sensitivity of HSV-1 and HSV-2 could depend on the peptide-targeted viral element, which may play a different role in the assembly and egress of the two viruses. Indeed, differences in the biological function of some viral proteins, conserved among *Alphaherpesvirinae*, were reported [43,44].

Interestingly, the peptide A-3302-B was previously reported as an antagonist of TRPV-1, a nonselective cation channel involved in the release of inflammatory mediators in the body, nociception, and temperature regulation [31,45,46]. Of note, several TRPV-1-induced events, such as the NF-κB activation, the proinflammatory cytokine production, and the remodelling of the microtubule cytoskeleton, are involved in HSV intracellular replicative cycle and pathogenesis [47,48,49,50,51,52]. Thus, we cannot exclude that the peptide’s antagonistic effect on TRPV-1 may be responsible, at least in part, for its anti-HSV-2 activity. However, further studies are required to clarify the possible involvement of the TRPV-1 signalling pathway in the peptide’s anti-HSV-2 activity.

Given its antiviral activity against ACV-resistant HSV-2 strains and the mechanism of action that differs from that of the approved drugs, A-3302-B could be a promising candidate to develop a therapeutic tool especially for clinical settings, in which the emergence of resistance to acyclovir determines cross-resistance to similar approved antiviral drugs and is associated with severe mucosal disease and systemic dissemination [53]. Moreover, it has been demonstrated that peptides as therapeutic agents possess various favourable characteristics for their clinical application, such as high specificity and effectiveness and peptidase biodegradability that limits the accumulation in tissues [24]. In this context, further studies are necessary to assess the antiviral activity and biocompatibility of the peptide in preclinical models in view of a clinical application.

## 3. Materials and Methods

### 3.1. Reagents

Methylcellulose, crystal violet, dimethyl sulfoxide (DMSO), sodium dodecyl-sulphate (SDS), NP-40, sodium deoxycholate, a cocktail of protease inhibitors, Tween 20, glycine, and Triton X-100 were purchased from Sigma-Aldrich (Saint Louis, MO, USA). The anti-HSV-2 ICP4 polyclonal antibody (ab96431) was from Abcam (Cambridge, UK). The anti-HSV-2 ICP8 monoclonal antibody (clone 4E6—H2A024) and the anti-HSV-1/2 gD monoclonal antibody (clone 2C10—HA025) were purchased from Virusys Corporation (Taneytown, MD, USA). The anti-actin antibody was from Millipore (Burlington, MA, USA) (clone C4—MAB1501R). The antibodies peroxidase-conjugated AffiniPure F(ab’)2 Fragment goat anti-mouse IgG (H + L) and goat anti-rabbit IgG (H + L) were from Jackson ImmunoResearch Laboratories Inc. (West Grove, PA, USA).

### 3.2. Cultivation of a Marine Bacterium Micromonospora sp. MAG 9-7 and Isolation of the Peptide A-3302-B

The marine bacterium *Micromonospora* sp. MAG 9-7, a rare actinomycete, was isolated from the marine sediment at Chonburi province, Thailand, at the latitude N12° 58′20.2″ and the longitude E100°54′05.8″. Taxonomic identification of the marine bacterium strain MAG 9-7 was by analysis of 16S rRNA gene sequence. The phylogenetic tree was constructed, and similarity comparison was performed for the 16S rDNA sequence of the marine bacterium using the sequences obtained from the GenBank database (https://www.ncbi.nlm.nih.gov/genbank, accessed on 2 September 2021). Phylogenetic tree analysis (Supplementary data, Figure S5) revealed that the marine bacterium strain MAG 9-7 is closely related to *Micromonospora* sp., and this bacterium was deposited at the Institute of Marine Science, Burapha University, Chonburi, Thailand.

The marine bacterium *Micromonospora* sp. MAG 9-7 was grown in an ISP2 medium using seawater instead of distilled water for the preparation of the medium. The ISP2 medium (in 1 L of seawater) is composed of 4 g of yeast extract, 10 g of malt extract powder, and 4 g of dextrose. The bacterium was grown in 1 L flasks, each containing 250 mL, under shaking conditions at 25 °C for 8 days. The total volume of 4.5 L of the culture was obtained, and it was extracted with ethyl acetate (EtOAc) 3 times (each with an equal volume of EtOAc), giving 3.0 g of a crude extract. Purification of the crude extract was by Sephadex LH-20 column chromatography (3 × 82 cm), using methanol (MeOH) as an eluent, which gave 11 fractions (F1–F11). The fraction F5 (308.9 mg) was washed with MeOH, giving insoluble material (167.5 mg) identified as the peptide A-3302-B (^1^H and ^13^C NMR spectra, as well as MS and MS/MS spectra, of A-3302-B are in the Supplementary data). The peptide A-3302-B exhibited a >96% purity based on ^1^H and ^13^C NMR analysis, and it was used for the antiviral assay.

### 3.3. Cell Lines and Viruses

African green monkey kidney cells (Vero, ATCC^®^ CCL-81) and low-passage-number (<30) human foreskin fibroblasts (HFF-1, ATCC^®^ SCRC-1041) were grown as monolayers in Dulbecco’s modified Eagle’s medium (DMEM) (Sigma-Aldrich), supplemented with 10% heat-inactivated fetal bovine serum (FBS) (Sigma-Aldrich) and with 1% (*v*/*v*) antibiotic–antimycotic solution (Zell Shield, Minerva Biolabs, Berlin, Germany) in humidified 5% CO_2_ atmosphere at 37 °C.

The neurovirulent strains LV and MS (ATCC^®^ VR-540) of HSV-1 and HSV-2, respectively, were propagated on Vero cells. The laboratory HSV-2 strain with phenotypic resistance to Acyclovir (HSV-2 ACV-r) was generated as described elsewhere [36]. The generation of viral stocks and viral titration by plaque assay were carried out on Vero cells, as described elsewhere [54]. As previously reported, a bacterial artificial chromosome-derived HCMV Towne strain, incorporating the green fluorescent protein (GFP) sequence, was propagated and titrated by fluorescence focus assay on HFF-1 cells [55].

### 3.4. Cell Viability Assay

Cell viability was measured using the MTS [3-(4,5-dimethylthiazol-2-yl)-5-(3-carboxymethoxyphenyl)-2-(4-sulfophenyl)-2H-tetrazolium] assay, as described elsewhere [56]. Treatment of control wells with equal volumes of DMSO was performed in order to rule out the possibility of any cytotoxic effect ascribable to the solvent. The effect on cell viability of A-3302-B at different concentrations was expressed as a percentage by comparing absorbances of treated cells with those of cells incubated with the control medium alone. The 50% cytotoxic concentrations (CC_50_) and 95% confidence intervals (CIs) were determined using GraphPad Prism 5.0 software (Graph-Pad Software, San Diego, CA, USA).

### 3.5. HSV Inhibition Assay

A plaque reduction assay was performed to evaluate the effect of A-3302-B on HSV infection. Vero cells preseeded in 24-well plates at a density of 10 × 10^4^ cells/well were incubated with increasing concentrations of the compound for 2 h at 37 °C. Subsequently, the serially diluted compound together with HSV-1, HSV-2, or acyclovir-resistant HSV-2 at MOI 0.001 PFU/cell (plaque-forming units per cell) was added to the cells, which were then incubated at 37 °C for 2 h. The virus inoculum was then removed, and the cells were washed and overlaid with DMEM medium containing 1.2% methylcellulose (Sigma-Aldrich) and serial dilutions of the compound. Control wells were subjected to the same protocol in the presence of equal volumes of DMSO instead of compound. After 24 h (HSV-2) or 48 h (HSV-1) of incubation at 37 °C, cells were fixed and stained with 0.1% crystal violet in 20% ethanol, and viral plaques were counted. The results were reported as the percentage of plaques in treated wells compared to the control wells. Where the peptide showed dose-dependent antiviral activity, half-maximal antiviral effective concentration (EC_50_) and concentration that reduced viral infectivity by 90% (EC_90_) values with 95% CIs were calculated by regression analysis using the software GraphPad Prism 5.0 (GraphPad Software, San Diego, CA, USA).

### 3.6. Time-of-Addition Assay

The assays evaluate the antiviral activity of the compound when administered before, during or after HSV-2 infection. Cells were incubated with different concentrations of A-3302-B (from 100 μM to 0.4 μM) in a 24-well plate at 37 °C for 2 h before infection (pretreatment), for 2 h during infection (cotreatment) or 24 h after removal of virus inoculum (post-treatment). Cells were concurrently infected with HSV-2 at MOI of 0.001 PFU/cell and treated for plaque reduction assay. Plaque size was measured with an Axiovert 200 inverted microscope (Zeiss, Jena, Germany) equipped with Infinity3 microscope camera (Lumenera, Ottawa, ON, Canada) and Infinity Capture software (Lumenera), and was analyzed with Infinity Analyze software (Lumenera) and ImageJ software (Bethesda, MD, USA).

### 3.7. HCMV Inhibition Assay

The anti-HCMV activity of the peptide was assessed via a focus reduction assay on confluent HFF-1 cells in 96-well plates. Cells were pretreated with serial dilutions of A-3302-B for 2 h at 37 °C. Subsequently, virus at MOI 0.01 FFU/cell (focus forming units per cell) and serially diluted compound were added to the cells and incubated for a further 2 h. The inocula were removed, and the cells were washed and overlaid with a 1.2% methylcellulose DMEM medium containing serial dilutions of the compound. Control wells were subjected to the same protocol in the presence of equal volumes of DMSO instead of compound. After five days of incubation at 37 °C, HCMV Towne-infected cells and foci were visualized as green fibroblasts using an inverted fluorescence microscope (Axiovert 200, Zeiss). Fluorescent foci were counted, and the percent inhibition of virus infectivity was determined by comparing the number of foci in treated wells with the number in control wells. EC_50_ and EC_90_ values and corresponding CIs were calculated as described above.

### 3.8. Immunoblotting

The ability of A-3302-B to inhibit the HSV-2 protein expression was evaluated via immunoblotting. Vero cells were infected with HSV-2 (MOI 1 PFU/cell) in the absence or presence of A-3302-B (EC_90_) or acyclovir (15 µM). Whole-cell extracts were prepared 4 h postinfection (hpi) by resuspending pelleted cells in lysis buffer containing 150 mM NaCl, 50 mM Tris-HCl (pH 8), 0.1% SDS, 1% NP-40, 0.5% sodium deoxycholate and a cocktail of protease inhibitors. Soluble proteins were collected by centrifugation at 15,000× *g*, quantified and stored at −80 °C as previously described [54]. For immunoblotting, proteins were separated by SDS-polyacrylamide gel electrophoresis (PAGE) and transferred to Immobilon-P membranes (Millipore). Membranes were then incubated overnight with blocking buffer consisting of 5% nonfat dry milk in 10 mM Tris-Cl (pH 7.5), 100 mM NaCl, 0.1% Tween 20 and immunostained with the anti-HSV-2 pAb against ICP4 protein, the anti-HSV-2 mAbs against ICP8 and gD proteins, and the anti-actin mAb. Immunocomplexes were detected using a secondary anti-rabbit or anti-mouse goat antibody conjugated to horseradish peroxidase and visualized using enhanced chemiluminescence (Super Signal™, Thermo Scientific, Waltham, MA, USA), according to the manufacturer’s instructions, with the ChemiDoc^TM^ Touch Imaging System (Bio-Rad Laboratories, Inc., Hercules, CA, USA).

### 3.9. Virus Inactivation Assay

The ability of A-3302-B to directly inactivate HSV-2 particles was investigated by mixing A-3302-B at EC_90_ dose and 10^5^ PFU of virus in a total volume of 100 µL and incubating it for 2 h at 37 °C. Subsequently, the mixture was serially diluted to the noninhibitory concentration of the peptide, and the residual viral infectivity was determined by viral titration.

### 3.10. Postinfection Kinetics Assay

The assay evaluates the antiviral activity of A-3302-B when administered at different time points after HSV-2 infection. Vero cells monolayers in 24-well plates were infected with HSV-2 at MOI of 0.001 PFU/cell for 2 h at 37 °C, followed by a gentle wash to remove the unbound virus. A-3302-B at a concentration corresponding to EC_90_ value was added at 0, 1, 2, 3, 4, 6, and 16 h postinfection in 1.2% methylcellulose DMEM medium and incubated at 37 °C for 24 h. After incubation, cells were fixed and stained with 0.1% crystal violet in 20% ethanol to count the number of viral plaques.

### 3.11. Virus Yield Reduction Assays

The peptide’s effect on the production of infectious viral particles was investigated by infecting Vero cells with HSV-2 at an MOI of 0.01 PFU/cells at 37 °C for 2 h and subsequently treating cells with serial dilutions of A-3302-B (from 100 μM to 0.4 μM). When control cultures displayed extensive cytopathology, cultures were harvested and pooled as appropriate 24 h after infection and cell-free virus infectivity titers were determined by plaque assay in Vero cell monolayers. The end-point of the assay was the effective concentration of compound that reduced virus yield by 50% (EC_50_) compared to untreated virus controls.

To evaluate the difference of viral titer of intracellular viral progeny and that released in the supernatant, a virus yield reduction assay was performed as described above, infecting with MOI of 0.01 PFU/cells and treating cells with compound at 100 μM. After 24 h, cells and supernatants were harvested and clarified separately and titrated accordingly by plaque assay on Vero cells. 

### 3.12. Attachment Assay

HSV-2 at MOI 0.01 PFU/cell was allowed to attach to prechilled confluent Vero cells in the presence of serial dilutions of A-3302-B (0.4 to 100 μM) for 2 h at 4 °C. Cells were then washed with a cold medium to remove the unbound virus and incubated for 2 h at 37 °C to allow virus entry. Outer virions were inactivated with acidic glycine (0.1 M, pH 3.0) for 1 min at room temperature. Cells were washed and treated as described above for the plaque reduction assay (Materials and Methods Section 3.5).

### 3.13. Entry Assay

The same protocol of the attachment assay was performed, but with the addition of A-3302-B during virus entry at 37 °C for 2 h.

### 3.14. Transmission Electron Microscopy

Pellet from infected and A-3302-B (100 µM) treated Vero cells were harvested at 16 hpi and fixed in 1% paraformaldehyde (Merck, Darmstadt, Germany), 1.25%glutaraldehyde (Fluka, St Louis, MO, USA), and 0.5% saccharose in 0.1 M Sörensen phosphate buffer (pH 7.2) for 2 h. For resin embedding, procedure samples were postfixed in 2% osmium tetroxide (SIC, Società Italiana Chimici, Rome, Italy) for 2 h and dehydrated in ethanol (Sigma Aldrich, St. Louis, MO, USA) from 30 to 100% (5 min each passage). After 2 passages of 7 min in propylene oxide, one passage of 1 h in a 1:1 mixture of propylene oxide (Sigma Aldrich) and Glauerts’ mixture of resins, samples were embedded in Glauerts’ mixture of resins (made of equal parts of Araldite M and the Araldite Harter, HY 964, Sigma Aldrich). In the resin mixture, 0.5% of the plasticizer dibutyl phthalate (Sigma Aldrich) was added. For the final step, 2% of accelerator 964 was added to the resin to promote resin polymerization at 60 °C. Ultrathin sections (70 nm thick) were cut using an Ultracut UCT ultramicrotome (Leica Microsystems, Wetzlar, Germany), stained with a solution of 4% UAR-EMS uranyl acetate replacement in distilled water, and analyzed using a JEM-1010 transmission electron microscope (JEOL, Tokyo, Japan) equipped with a Mega-View-III digital camera and a Soft-Imaging-System (SIS, Münster, Germany) for the computerized acquisition of the images.

### 3.15. Statistical Analysis

All data were analyzed with GraphPad Prism version 5.00 software. Results were expressed as mean values for three independent experiments performed in duplicate. The EC_50_ and EC_90_ values for inhibition curves were calculated by regression analysis by fitting a variable slope-sigmoidal dose–response curve. The Student’s T-test was used to compare viral titers in virus inactivation assay and viral titers intracellular and supernatant in the virus yield reduction assay. The viral titers of control and treated samples in virus yield reduction assay and the measurements of plaque size in the presence and absence of the compound were compared using a one-way analysis of variance (ANOVA) followed by a Bonferroni test. Significance was reported for *p* value <0.05 (*), <0.01 (**) and <0.001 (***).

## 4. Conclusions

Overall, this study discloses the anti-HSV-2 of the bacterial-derived peptide A-3302-B. By specific assays, we identified the egress of virions from infected cells, a late step of the viral replicative cycle, as the major event inhibited by the peptide. Moreover, we demonstrated its activity against an ACV-resistant strain. These features, along with the previously reported anti-inflammatory activity [31], suggest that the peptide A-3302-B may be a promising candidate for developing new therapeutic options to treat genital HSV-2 infections resistant to conventional therapies.

## Figures and Tables

**Table 1:** Comparison of HSV-2 titer intracellularly and in the supernatant after treatment with A-3302-B.

Treatment	Sample	Viral Titer (Mean) (PFU)	Viral Titer (Log_10_)
Untreated	Cells	1.14 × 10^5^	5.06
	Supernatant	1.01 × 10^3^	3.01
A-3302-B	Cells	1.26 × 10^4^	4.10
	Supernatant	6.00 × 10^0^	0.78

## Data Availability

Taxonomic identification of the marine bacterium strain MAG 9-7 was by analysis of 16S rRNA gene sequence, using the sequences obtained from the GenBank database (https://www.ncbi.nlm.nih.gov/genbank, accessed on 2 September 2021).

## Supplementary Materials

The following supporting information can be downloaded at: https://www.mdpi.com/article/10.3390/ijms23020947/s1.

## References

[1] WHO (World Health Organization) Herpes Simplex Virus. https://www.who.int/news-room/fact-sheets/detail/herpes-simplex-virus.

[2] James C., Harfouche M., Welton N.J., Turner K.M.E., Abu-Raddad L.J., Gottlieb S.L., Looker K.J. (2020). Herpes simplex virus: Global infection prevalence and incidence estimates, 2016. Bull. World Health Organ..

[3] Jonsson M.K., Wahren B. (2004). Sexually transmitted herpes simplex viruses. Scand. J. Infect. Dis..

[4] Choudhry S., Ramachandran V.G., Das S., Bhattacharya S.N., Mogha N.S. (2010). Pattern of sexually transmitted infections and performance of syndromic management against etiological diagnosis in patients attending the sexually transmitted infection clinic of a tertiary care hospital. Indian J. Sex. Transm. Dis..

[5] Van De Laar M.J.W., Termorshuizen F., Slomka M.J., Van Doornum G.J.J., Ossewaarde J.M., Brown D.W.G., Coutinho R.A., Van Den Hoek J.A.R. (1998). Prevalence and correlates of herpes simplex virus type 2 infection: Evaluation of behavioural risk factors. Int. J. Epidemiol..

[6] Groves M.J. (2016). Genital herpes: A review. Am. Fam. Physician.

[7] Looker K.J., Welton N.J., Sabin K.M., Dalal S., Vickerman P., Turner K.M.E., Boily M., Gottlieb S.L. (2019). Global and regional estimates of the contribution of herpes simplex virus type 2 infection to HIV incidence: A population attributable fraction analysis using published epidemiological data. Lancet Infect. Dis..

[8] Cytomegalovirus (CMV) and Congenital CMV Infection|CDC. https://www.cdc.gov/cmv/.

[9] Brunnemann A., Hoffmann A., Deinhardt-Emmer S., Nagel C., Rose R., Fickenscher H., Sauerbrei A., Krumbholz A. (2018). Relevance of non-synonymous thymidine kinase mutations for antiviral resistance of recombinant herpes simplex virus type 2 strains. Antivir. Res..

[10] Silva D., Cardoso J., Silva S., Arruda L., Medeiros R., Moraes M., Sousa R. (2018). HCMV UL97 phosphotransferase gene mutations may be associated with antiviral resistance in immunocompromised patients in Belém, PA, Northern Brazil. Rev. Soc. Bras. Med. Trop..

[11] Danve-Szatanek C., Aymard M., Thouvenot D., Morfin F., Agius G., Bertin I., Billaudel S., Chanzy B., Coste-Burel M., Finkielsztejn L. (2004). Surveillance Network for Herpes Simplex Virus Resistance to Antiviral Drugs: 3-Year Follow-Up. J. Clin. Microbiol..

[12] Mahendran A.S.K., Lim Y.S., Fang C.-M., Loh H.-S., Le C.F. (2020). The Potential of Antiviral Peptides as COVID-19 Therapeutics. Front. Pharmacol..

[13] Chowdhury A., Reehl S., Kehn-Hall K., Bishop B., Webb-Robertson B. (2020). Better understanding and prediction of antiviral peptides through primary and secondary structure feature importance. Sci. Rep..

[14] Frecer V., Miertus S. (2020). Antiviral agents against COVID-19: Structure-based design of specific peptidomimetic inhibitors of SARS-CoV-2 main protease. RSC Adv..

[15] Pen G., Yang N., Teng D., Mao R., Hao Y., Wang J. (2020). A Review on the Use of Antimicrobial Peptides to Combat Porcine Viruses. Antibiotics.

[16] Maiti B.K. (2020). Potential Role of Peptide-Based Antiviral TherapyAgainst SARS-CoV-2 Infection. ACS Pharmacol. Transl. Sci..

[17] Matthews T., Salgo M., Greenberg M., Chung J., DeMasi R., Bolognesi D. (2004). Enfuvirtide: The first therapy to inhibit the entry of HIV-1 into host CD4 lymphocytes. Nat. Rev. Drug Discov..

[18] Butt A.A., Kanwal F. (2012). Boceprevir and telaprevir in the management of hepatitis C virus-infected patients. Clin. Infect. Dis..

[19] Agarwal G., Gabrani R. (2021). Antiviral Peptides: Identification and Validation. Int. J. Pept. Res. Ther..

[20] Qiao J., Li Y.-S., Zeng R., Liu F.-L., Luo R.-H., Huang C., Wang Y.-F., Zhang J., Quan B., Shen C. (2021). SARS-CoV-2 Mpro inhibitors with antiviral activity in a transgenic mouse model. Science.

[21] Newman D.J., Cragg G.M. (2020). Natural Products as Sources of New Drugs over the Nearly Four Decades from January 1981 to September 2019. J. Nat. Prod..

[22] Fjell C.D., Hiss J.A., Hancock R.E.W., Schneider G. (2012). Designing antimicrobial peptides: Form follows function. Nat. Rev. Drug Discov..

[23] Rodrigues T., Reker D., Schneider P., Schneider G. (2016). Counting on natural products for drug design. Nat. Chem..

[24] Vilas Boas L.C.P., Campos M.L., Berlanda R.L.A., de Carvalho Neves N., Franco O.L. (2019). Antiviral peptides as promising therapeutic drugs. Cell. Mol. Life Sci..

[25] Qi S., Gui M., Li H., Yu C., Li H., Zeng Z., Sun P. (2020). Secondary Metabolites from Marine *Micromonospora*: Chemistry and Bioactivities. Chem. Biodivers..

[26] Romero F., Espliego F., Baz J.P., De Quesada T.G., Grávalos D., De La Calle F., Fernández-Puentes J.L. (1997). Thiocoraline, a new depsipeptide with antitumor activity produced by a marine *Micromonospora*. I. Taxonomy, fermentation, isolation, and biological activities. J. Antibiot..

[27] Chen L., Zhao W., Jiang H.L., Zhou J., Chen X.M., Lian Y.Y., Jiang H., Lin F. (2018). Rakicidins G-I, cyclic depsipeptides from marine *Micromonospora chalcea* FIM 02-523. Tetrahedron.

[28] Kitajima Y., Waki M., Shoji J., Ueno T., Izumiya N. (1990). Revised structure of the peptide lactone antibiotic, TL-119 and/or A-3302-B. FEBS Lett..

[29] Chalasani A.G., Roy U., Nema S. (2018). Purification and characterisation of a novel antistaphylococcal peptide (ASP-1) from Bacillus sp. URID 12.1. Int. J. Antimicrob. Agents.

[30] Nakagawa Y., Nakazawa T., Shoji J. (1975). On the structure of a new antibiotic TL-119 (studies on antibiotics from the genus Bacillus. VI). J. Antibiot..

[31] Lin Z., Reilly C.A., Antemano R., Hughen R.W., Marett L., Concepcion G.P., Haygood M.G., Olivera B.M., Light A., Schmidt E.W. (2011). Nobilamides A-H, long-acting transient receptor potential vanilloid-1 (TRPV1) antagonists from mollusk-associated bacteria. J. Med. Chem..

[32] Schmidt E., Light A.R., Olivera B.M., Reilly C.A., Lin Z., Concepcion G.P. Antagonists of TRPV1 Receptor. https://patentscope2.wipo.int/search/en/detail.jsf?docId=WO2012149218.

[33] Leary J.J., Wittrock R., Sarisky R.T., Weinberg A., Levin M.J. (2002). Susceptibilities of herpes simplex viruses to penciclovir and acyclovir in eight cell lines. Antimicrob. Agents Chemother..

[34] Boas L.C.P.V., de Lima L.M.P., Migliolo L., Mendes G.D.S., de Jesus M.G., Franco O.L., Silva P.A. (2017). Linear antimicrobial peptides with activity against herpes simplex virus 1 and Aichi virus. Biopolymers.

[35] Liang X., Nong X.H., Huang Z.H., Qi S.H. (2017). Antifungal and Antiviral Cyclic Peptides from the Deep-Sea-Derived Fungus *Simplicillium obclavatum* EIODSF 020. J. Agric. Food Chem..

[36] Ghosh M., Civra A., Rittà M., Cagno V., Mavuduru S.G., Awasthi P., Lembo D., Donalisio M. (2016). Ficus religiosa L. bark extracts inhibit infection by herpes simplex virus type 2 in vitro. Arch. Virol..

[37] Galdiero S., Falanga A., Tarallo R., Russo L., Galdiero E., Cantisani M., Morelli G., Galdiero M. (2013). Peptide inhibitors against herpes simplex virus infections. J. Pept. Sci..

[38] Roy M., Lebeau L., Chessa C., Damour A., Ladram A., Oury B., Boutolleau D., Bodet C., Lévêque N. (2019). Comparison of anti-viral activity of frog skin anti-microbial peptides temporin-sha and [K^3^]SHa to LL-37 and temporin-Tb against herpes simplex virus type 1. Viruses.

[39] Zeng Z., Zhang R., Hong W., Cheng Y., Wang H., Lang Y., Ji Z., Wu Y., Li W., Xie Y. (2018). Histidine-rich modification of a scorpion-derived peptide improves bioavailability and inhibitory activity against HSV-1. Theranostics.

[40] Diamond G., Molchanova N., Herlan C., Fortkort J.A., Lin J.S., Figgins E., Bopp N., Ryan L.K., Chung D., Adcock R.S. (2021). Potent antiviral activity against HSV-1 and SARS-CoV-2 by antimicrobial peptoids. Pharmaceuticals.

[41] Marcocci M.E., Amatore D., Villa S., Casciaro B., Aimola P., Franci G., Grieco P., Galdiero M., Palamara A.T., Mangoni M.L. (2018). The amphibian antimicrobial peptide temporin b inhibits in vitro herpes simplex virus 1 infection. Antimicrob. Agents Chemother..

[42] Draganova E.B., Heldwein E.E. (2021). Virus-derived peptide inhibitors of the herpes simplex virus type 1 nuclear egress complex. Sci. Rep..

[43] Gao J., Yan X., Banfield B.W. (2018). Comparative Analysis of UL16 Mutants Derived from Multiple Strains of Herpes Simplex Virus 2 (HSV-2) and HSV-1 Reveals Species-Specific Requirements for the UL16 Protein. J. Virol..

[44] Shindo K., Kato A., Koyanagi N., Sagara H., Arii J., Kawaguchi Y. (2016). Characterization of a Herpes Simplex Virus 1 (HSV-1) Chimera in Which the Us3 Protein Kinase Gene Is Replaced with the HSV-2 Us3 Gene. J. Virol..

[45] Du Q., Liao Q., Chen C., Yang X., Xie R., Xu J. (2019). The Role of Transient Receptor Potential Vanilloid 1 in Common Diseases of the Digestive Tract and the Cardiovascular and Respiratory System. Front. Physiol..

[46] Gavva N.R. (2008). Body-temperature maintenance as the predominant function of the vanilloid receptor TRPV1. Trends Pharmacol. Sci..

[47] Kong W.L., Peng Y.Y., Peng B.W. (2017). Modulation of neuroinflammation: Role and therapeutic potential of TRPV1 in the neuro-immune axis. Brain Behav. Immun..

[48] Yoshida A., Furube E., Mannari T., Takayama Y., Kittaka H., Tominaga M., Miyata S. (2016). TRPV1 is crucial for proinflammatory STAT3 signaling and thermoregulation-associated pathways in the brain during inflammation. Sci. Rep..

[49] Goswami C., Hucho T. (2008). Submembraneous microtubule cytoskeleton: Biochemical and functional interplay of TRP channels with the cytoskeleton. FEBS J..

[50] Lu X., Huang C., Zhang Y., Lin Y., Wang X., Li Q., Liu S., Tang J., Zhou L. (2017). The Us2 Gene Product of Herpes Simplex Virus 2 modulates NF-κB activation by targeting TAK1. Sci. Rep..

[51] Azher T.N., Yin X.T., Stuart P.M. (2017). Understanding the role of chemokines and cytokines in experimental models of herpes simplex keratitis. J. Immunol. Res..

[52] Lyman M.G., Enquist L.W. (2009). Herpesvirus Interactions with the Host Cytoskeleton. J. Virol..

[53] Piret J., Boivin G. (2011). Resistance of herpes simplex viruses to nucleoside analogues: Mechanisms, prevalence, and management. Antimicrob. Agents Chemother..

[54] Donalisio M., Quaranta P., Chiuppesi F., Pistello M., Cagno V., Cavalli R., Volante M., Bugatti A., Rusnati M., Ranucci E. (2016). The AGMA1 poly(amidoamine) inhibits the infectivity of herpes simplex virus in cell lines, in human cervicovaginal histocultures, and in vaginally infected mice. Biomaterials.

[55] Donalisio M., Rittà M., Tonetto P., Civra A., Coscia A., Giribaldi M., Cavallarin L., Moro G.E., Bertino E., Lembo D. (2018). Anti-cytomegalovirus activity in human milk and colostrum from mothers of preterm infants. J. Pediatr. Gastroenterol. Nutr..

[56] Milani M., Donalisio M., Bonotto R.M., Schneider E., Arduino I., Boni F., Lembo D., Marcello A., Mastrangelo E. (2021). Combined in silico and in vitro approaches identified the antipsychotic drug lurasidone and the antiviral drug elbasvir as SARS-CoV2 and HCoV-OC43 inhibitors. Antivir. Res..

