# Supplementary material for: The Peptide A-3302-B Isolated from a Marine Bacterium Micromonospora sp. Inhibits HSV-2 Infection by Preventing the Viral Egress from Host Cells
Source: Int J Mol Sci. 2022 Jan 15;23(2):947. doi: 10.3390/ijms23020947 (PMC8778767; doi:10.3390/ijms23020947)
Supplement: Supplementary file 1 [file ijms-23-00947-s001.zip › ijms-1537513-supplementary.pdf]

Supplementary Materials

The peptide A-3302-B isolated from a marine bacterium *Micromonospora* sp. inhibits HSV-2 infection by preventing the viral egress from host cells

Figure S1: ^{1}H NMR spectrum of peptide A-3302-B in DMSO-d_6

Figure S2: ^{13}C NMR spectrum of peptide A-3302-B in DMSO-d_6

Figure S3: MS spectrum of peptide A-3302-B

Figure S4: MS/MS spectrum of peptide A-3302-B and its tentative MS fragmentation

Figure S5: Phylogenetic tree for similarity comparison of the marine bacterium MAG 9-7 among the other *Bacillus* and *Micromonospora* species

Figure S6: Effect of A-3302-B on the viability of Vero cells.

Figure S7: Virucidal activity of A-3302-B against HSV-2

Figure S8: Effect of A-3302-B on HSV-2 attachment and entry into host cells

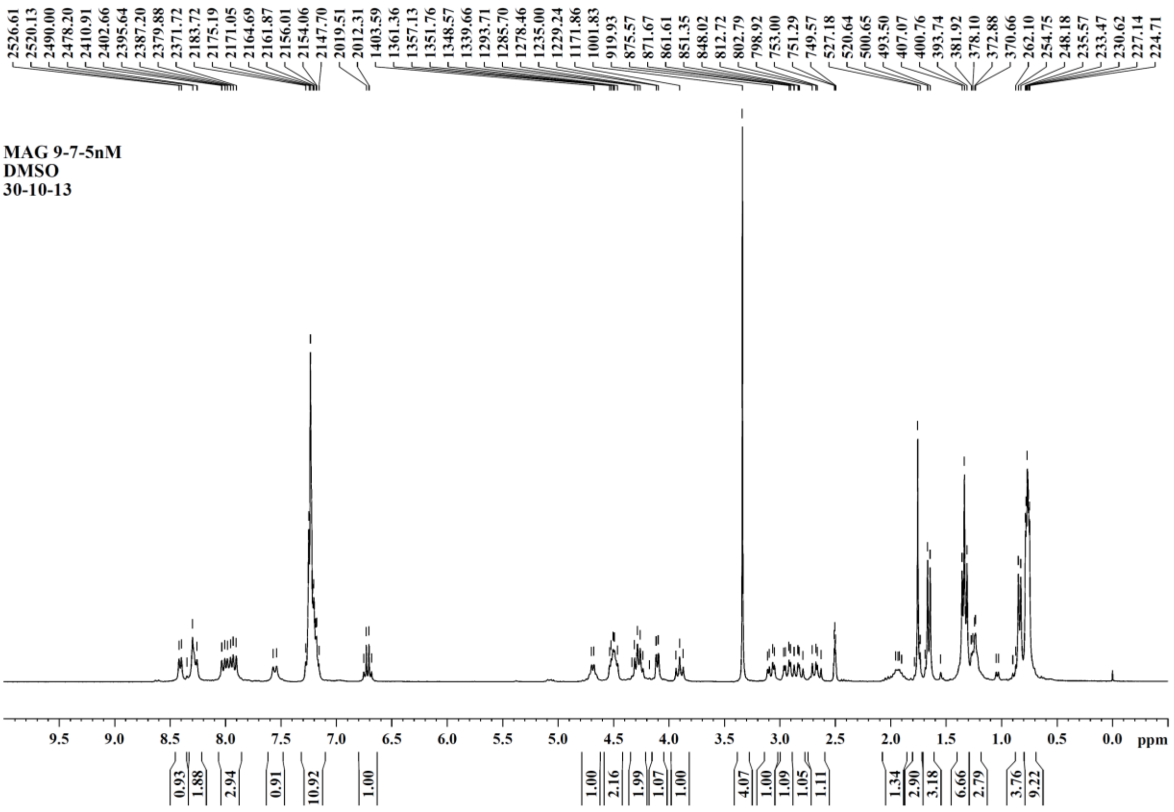

Figure S1. ^{1}H NMR spectrum of peptide A-3302-B in DMSO-d_6

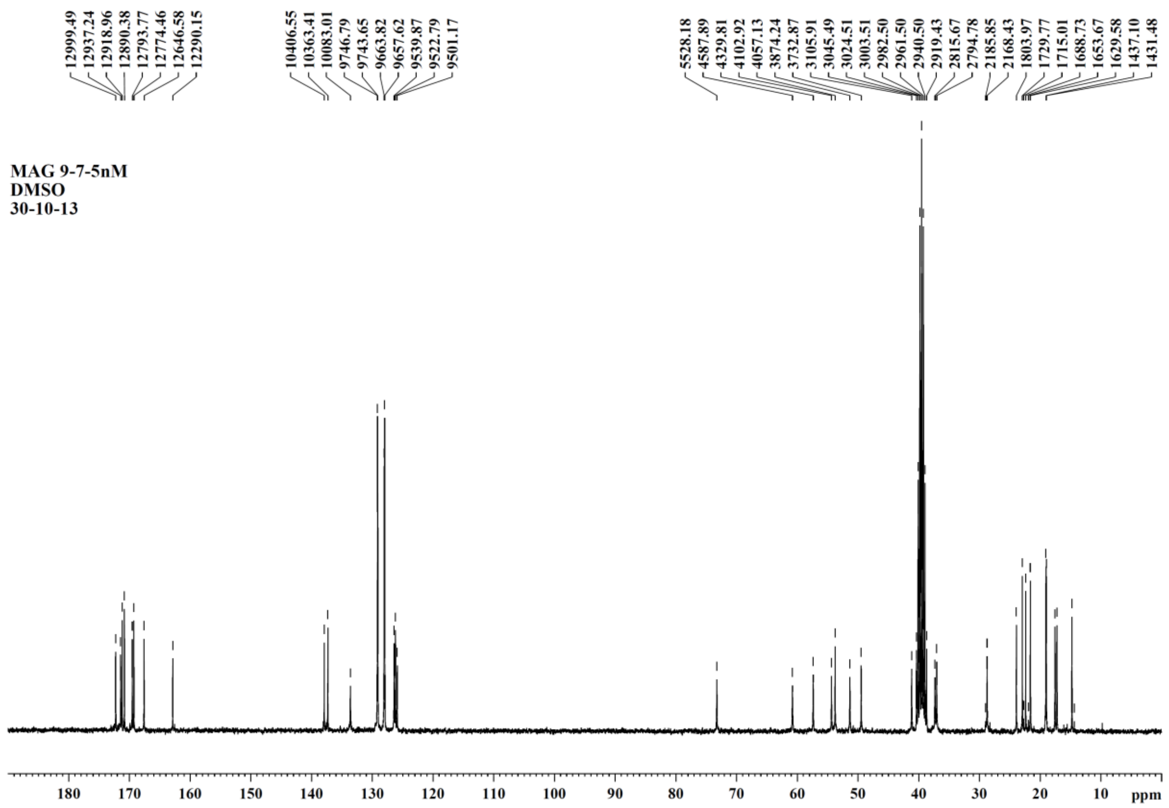

Figure S2. ^{13}C NMR spectrum of peptide A-3302-B in DMSO-d_6

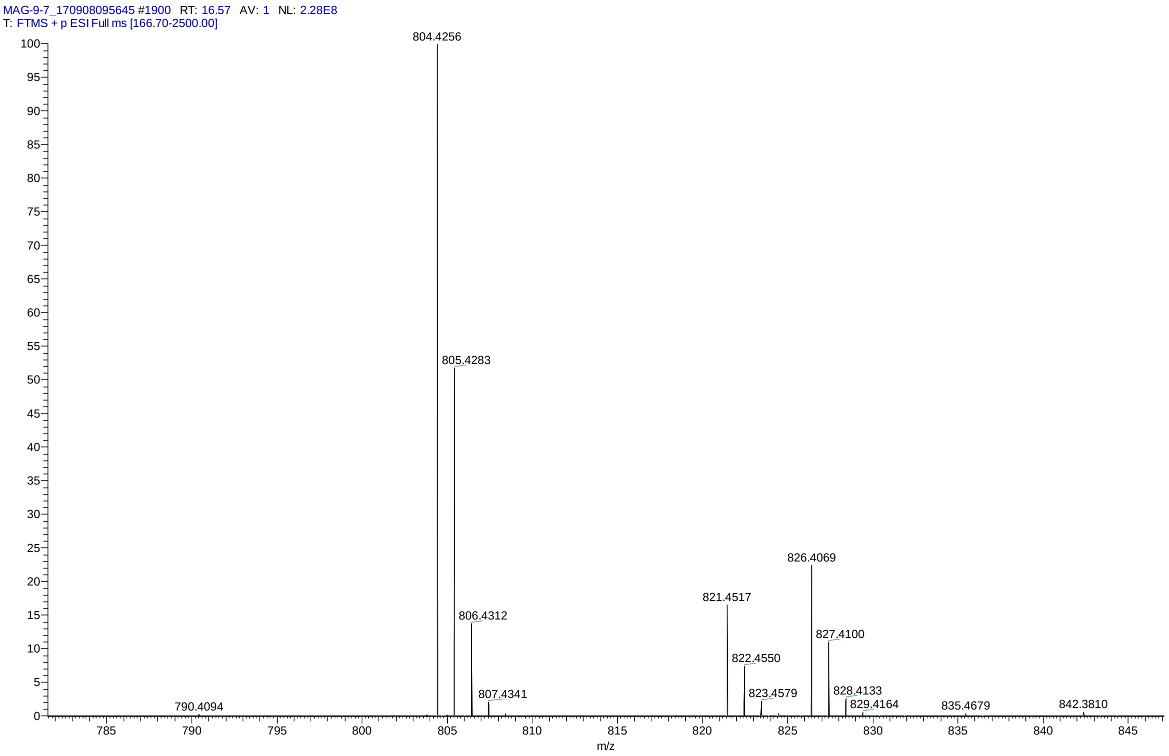

Figure S3. MS spectrum of peptide A-3302-B

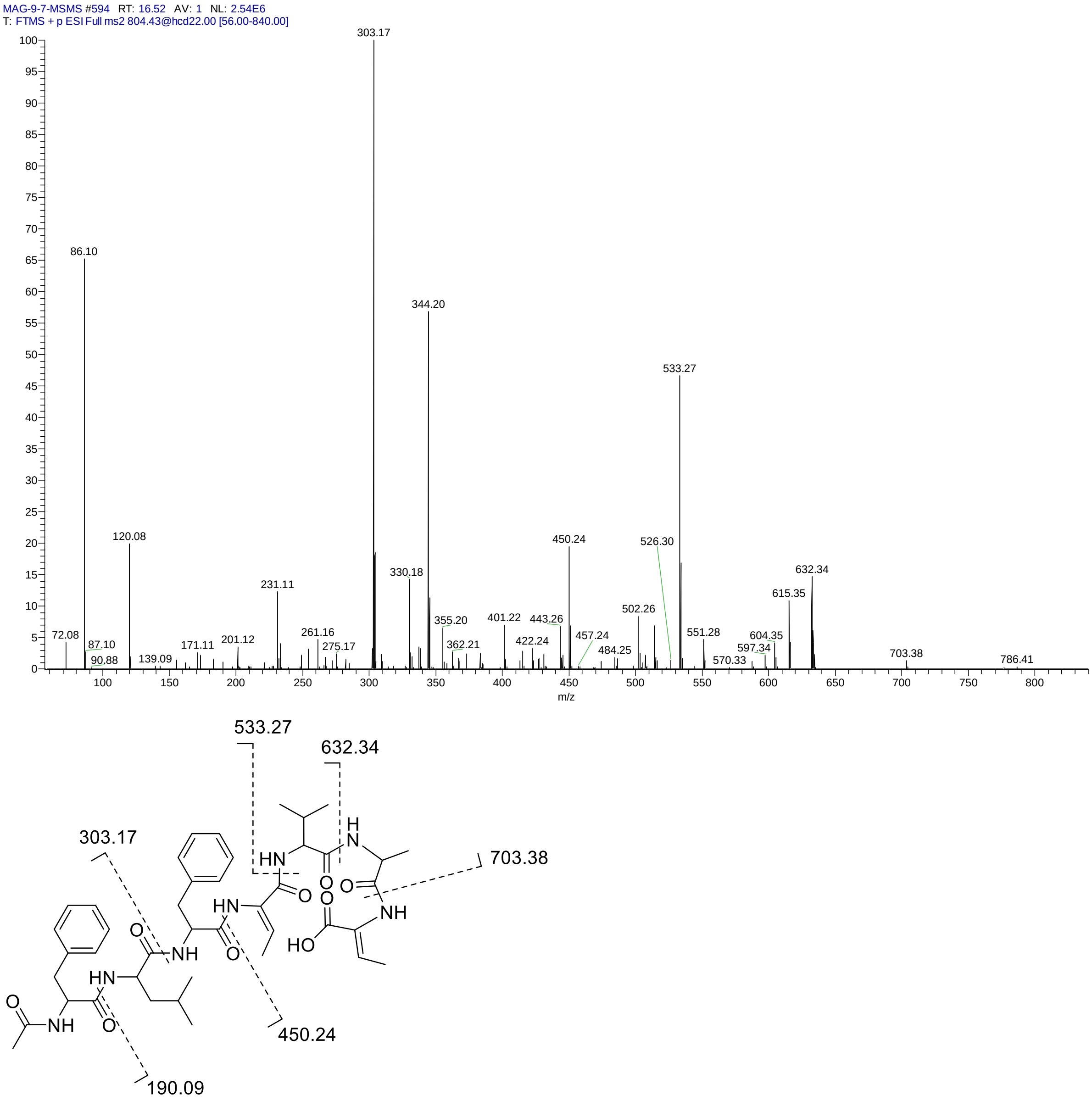

Figure S4. MS/MS spectrum of peptide A-3302-B and its tentative MS fragmentation.

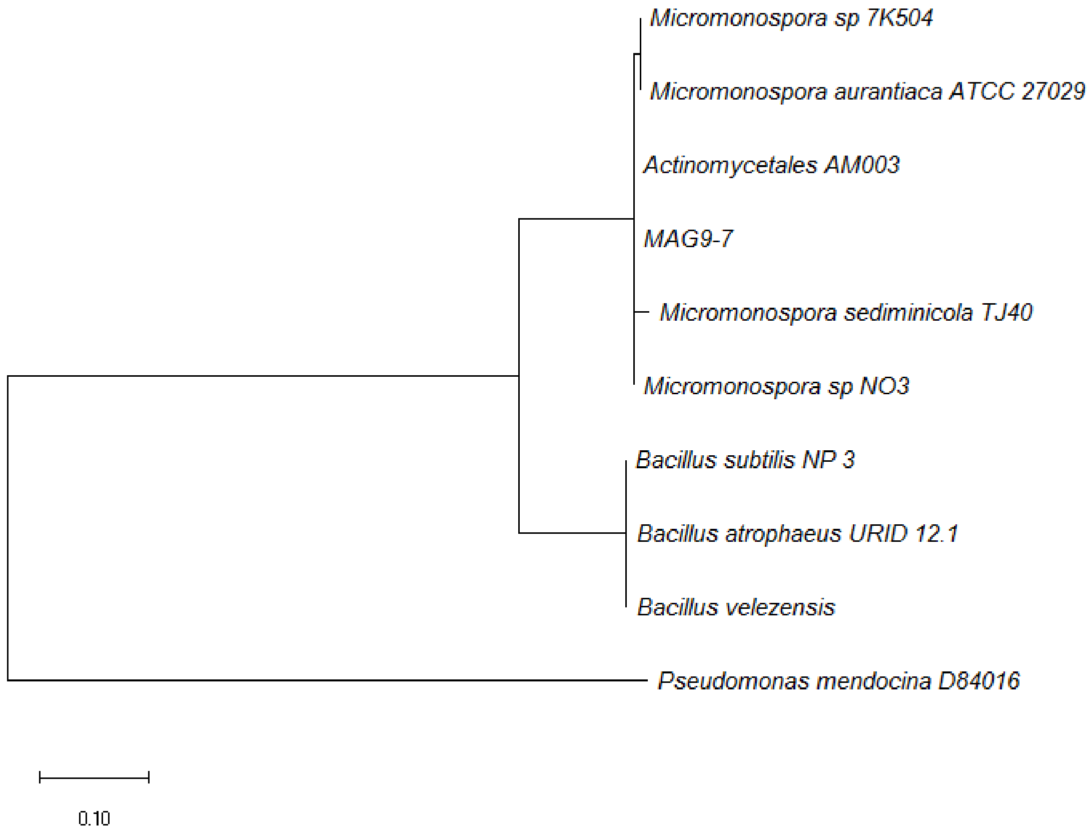

Figure S5. Phylogenetic tree for similarity comparison of the marine bacterium MAG 9-7 among the other *Bacillus* and *Micromonospora* species. *Pseudomonas mendocina* was included in phylogenetic tree as an outgroup species.

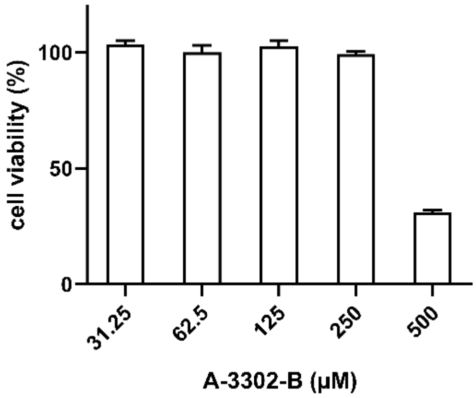

Figure S6. Effect of A-3302-B on the viability of non-infected Vero cells as a function of the drug concentration at 24 h. X axis: A-3302-B concentration (μM); Y axis: cell viability (% of DMSO-treated control). Error bars represent SEM for three independent experiments.

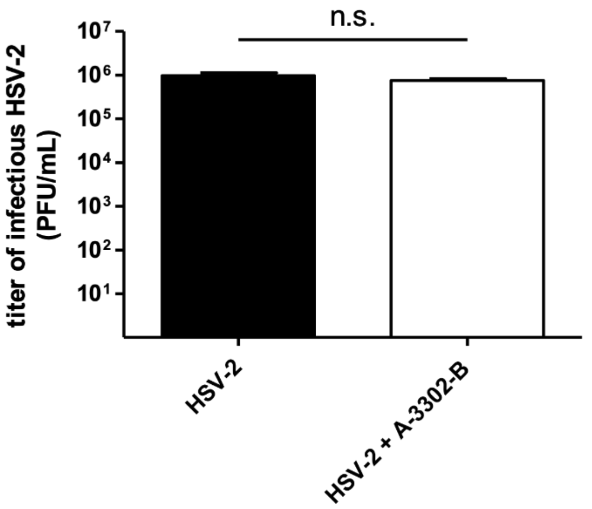

Figure S7. Virucidal activity of A-3302-B against HSV-2. Inactivation of HSV-2 particles by A-3302-B was evaluated incubating 10^5 PFU of HSV-2 for 2 h at 37° C with a concentration of compound that reduces the virus infection almost completely (EC_{90}). Subsequently, residual viral infectivity was determined by plaque assay. On y-axis, the infectious titers are expressed as plaque forming units per mL (PFU/mL). Error bars represent SEM for three independent experiments. Student's t-test was used to compare viral titers (n.s.: not significant).

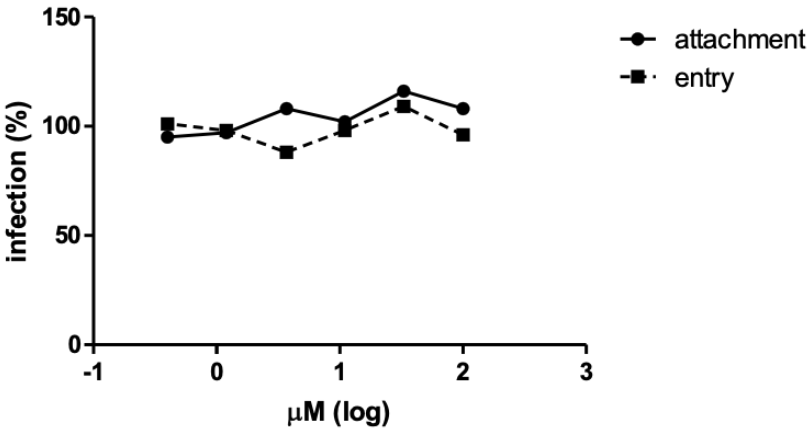

Figure S8. Effect of A-3302-B on HSV-2 attachment and entry into host cells. Plaque reduction assays were performed to evaluate the ability of compound to inhibit the attachment of virus to the cell membrane (attachment assay) or the entry of virus into cells (entry assay), as described in the Materials and Methods section. The percentage infection was calculated by comparing treated and untreated wells. Results are reported as mean and SEM.
